# Draft Genome Sequences of Two Clinical Mastitis-Associated Escherichia coli Strains, of Sequence Type 101 and Novel Sequence Type 13054, Isolated from Dairy Cows in Bangladesh

**DOI:** 10.1128/mra.00166-23

**Published:** 2023-07-10

**Authors:** Mohammad H. Rahman, Mohamed E. El Zowalaty, Linda Falgenhauer, Mohammad Ferdousur Rahman Khan, Jahangir Alam, Najmun Nahar Popy, M. Bahanur Rahman

**Affiliations:** a Department of Microbiology and Hygiene, Bangladesh Agricultural University, Mymensingh, Bangladesh; b Veterinary Medicine and Food Security Research Group, Medical Laboratory Sciences Program, Faculty of Health Sciences, Abu Dhabi Women's Campus, Higher Colleges of Technology, Abu Dhabi, United Arab Emirates; c German Center for Infection Research, Site Giessen-Marburg-Langen, Giessen, Germany; d Institute of Hygiene and Environmental Medicine, Justus Liebig University Giessen, Giessen, Germany; e Hessian University Competence Center for Hospital Hygiene, Justus Liebig University Giessen, Giessen, Germany; f National Institute of Biotechnology, Savar, Dhaka, Bangladesh; University of Maryland School of Medicine

## Abstract

Here, we report the draft genome sequences of two Escherichia coli strains that were isolated from raw milk samples obtained from lactating cows with mastitis in Bangladesh. One strain was assigned to a novel sequence type 13054, and the other strain belonged to sequence type 101.

## ANNOUNCEMENT

Mastitis is a complex, multi-etiological disease caused by more than 140 bacterial species. It is one of the most widespread diseases of major economic importance in the dairy industry worldwide (1, 2). Escherichia coli is one of the leading mastitis-causing pathogens (2–5). Mastitis is a disease with serious zoonotic potential associated with shedding of bacteria and their toxins, including Shiga toxin-producing E. coli strains (6), through unpasteurized milk, constituting potential threats to humans and other animals. Here, we report the draft genome sequences of two E. coli strains that were isolated from raw milk samples obtained from lactating cows with mastitis in Bangladesh. The study was performed due to the lack of genome-based studies of mastitis-causing E. coli strains from Bangladesh.

Mastitis milk samples (10 mL) that had been collected in sterile tubes from 36-month and 46-month-old lactating Holstein-Friesian cows (*Bos taurus taurus*) were transported to the laboratory, maintaining a proper cold chain. Nutrient broth (10 mL) was inoculated with 500 μL of a milk sample, incubated at 37°C for 18 h, and subsequently streaked on selective eosin methylene blue (EMB) agar plates (HiMedia Laboratories LLC., Mumbai, India). After incubation at 37°C for 24 h, pure single colonies were identified as E. coli using Gram staining and the indole, methyl red, Voges-Proskauer, citrate utilization (IMViC) test. The purified cultures of the two E. coli strains were submitted to Invent Technology Ltd. (Banani, Dhaka, Bangladesh) for whole genome sequencing. DNA was extracted from overnight cultures that had been grown in nutrient broth at 37°C, using a genomic DNA purification kit (Promega, WI, USA). Sequencing libraries were prepared using the Nextera XT library preparation kit (Illumina, CA, USA) and were sequenced on an Illumina NextSeq 550 system using the NextSeq 500/550 high-output kit v2.5 (300 cycles).

Default parameters were used for all software unless otherwise specified. Quality control was performed using ASA^3^P v1.4.0 (7). Assembly was performed using SPAdes v3.13.0 (8) integrated in ASA^3^P. Contigs, excluding those smaller than 200 bp, were uploaded to the National Center for Biotechnology Information (NCBI) (Bethesda, MD, USA) and annotated using the NCBI Prokaryotic Genome Annotation Pipeline (PGAP) v6.0 (9). Sequencing, assembly, and annotation data for BR-MHR261 and BR-MHR268 are summarized in Table 1.

**TABLE 1:** Basic characteristics of the whole-genome sequencing, assembly, and annotation of strains BR-MHR261 and BR-MHR268 reported in the present study

Parameter	Finding for strain:	
	BR-MHR261	BR-MHR268
No. of raw reads	17,971,212	19,625,988
Avg read length (nucleotides)	128.9	129
Avg coverage (×)	462	518
No. of contigs of >200 bp	93	152
*N*_50_ (bp)	212,731	217,132
Genome size (bp)	5,014,182	4,887,493
G+C content (%)	50.6	50.6
Total no. of genes	4,929	4,831
No. of coding sequences (with protein)	4,701	4,586
No. of RNA genes	87	86
No. of rRNAs (5S, 16S, 23S)	1, 1, 2	0, 2, 1
No. of complete rRNAs (5S, 16S, 23S)	1, 1, 0	0, 0, 1
No. of partial rRNAs (5S, 16S, 23S)	0, 0, 1	0, 2, 1
No. of tRNAs	75	74
No. of noncoding RNAs	8	9
No. of pseudogenes (total)	142	159

No antibiotic resistance genes were detected using ResFinder v4.0 (10). Virulence genes detected with the Virulence Factor Database (VFDB) integrated in ASA^3^P differed in the two isolates. BR-MHR261 and BR-MHR268 harbored 46 and 34 virulence determinants, respectively. Both isolates harbored genes involved in iron acquisition (enterobactin) and the E. coli common pilus. Unique features were type II secretion system genes in BR-MHR261 and type I fimbria genes in BR-MHR268.

Multilocus sequence typing (MLST) sequence types (STs) were determined using PubMLST (11) and the Achtman scheme (12). BR-MHR261 had a new and very rare ST (ST-13054). BR-MHR268 was an ST-101 isolate. E. coli ST-101 strains have already been detected in mastitis samples (13).

This report highlights the significance of continued genomic surveillance of mastitis-associated E. coli in food chain cattle and food production, which will help us to understand its role in the spread of antimicrobial resistance and pathogenesis.

### Data availability.

This whole-genome sequencing project has been deposited in DDBJ/ENA/GenBank under the accession numbers JALBGL000000000 (BR-MHR261) and JALBGK000000000 (BR-MHR268). The versions described here are the first versions. The sequences are publicly available under BioProject accession number PRJNA716986, BioSample accession numbers SAMN26025966 (BR-MHR261) and SAMN26025967 (BR-MHR268), and SRA accession numbers SRR18182111 (BR-MHR261) and SRR18182110 (BR-MHR268). All genomes are also publicly available at PubMLST (https://pubmlst.org/organisms/escherichia-spp).

## References

[1] Goulart DB, Mellata M. 2022. *Escherichia coli* mastitis in dairy cattle: etiology, diagnosis, and treatment challenges. Front Microbiol 13:928346. doi:10.3389/fmicb.2022.928346. 35875575 PMC9301288

[2] Pascu C, Herman V, Iancu I, Costina L. 2022. Etiology of mastitis and antimicrobial resistance in dairy cattle farms in the western part of Romania. Antibiotics 11:57. doi:10.3390/antibiotics11010057. 35052934 PMC8772981

[3] Al-Harbi H, Ranjbar S, Moore RJ, Alawneh JI. 2021. Bacteria isolated from milk of dairy cows with and without clinical mastitis in different regions of Australia and their AMR profiles. Front Vet Sci 8:743725. doi:10.3389/fvets.2021.743725. 34805335 PMC8600363

[4] Cheng WN, Han SG. 2020. Bovine mastitis: risk factors, therapeutic strategies, and alternative treatments: a review. Asian-Australas J Anim Sci 33:1699–1713. doi:10.5713/ajas.20.0156. 32777908 PMC7649072

[5] Dalanezi FM, Joaquim SF, Guimarães FF, Guerra ST, Lopes BC, Schmidt EMS, Cerri RLA, Langoni H. 2020. Influence of pathogens causing clinical mastitis on reproductive variables of dairy cows. J Dairy Sci 103:3648–3655. doi:10.3168/jds.2019-16841. 32089296

[6] Murinda SE, Ibekwe AM, Rodriguez NG, Quiroz KL, Mujica AP, Osmon K. 2019. Shiga toxin-producing *Escherichia coli* in mastitis: an international perspective. Foodborne Pathog Dis 16:229–243. doi:10.1089/fpd.2018.2491. 30624967

[7] Schwengers O, Hoek A, Fritzenwanker M, Falgenhauer L, Hain T, Chakraborty T, Goesmann A. 2020. ASA^3^P: an automatic and scalable pipeline for the assembly, annotation and higher-level analysis of closely related bacterial isolates. PLoS Comput Biol 16:e1007134. doi:10.1371/journal.pcbi.1007134. 32134915 PMC7077848

[8] Prjibelski A, Antipov D, Meleshko D, Lapidus A, Korobeynikov A. 2020. Using SPAdes de novo assembler. Curr Protoc Bioinformatics 70:e102. doi:10.1002/cpbi.102. 32559359

[9] Tatusova T, DiCuccio M, Badretdin A, Chetvernin V, Nawrocki EP, Zaslavsky L, Lomsadze A, Pruitt KD, Borodovsky M, Ostell J. 2016. NCBI Prokaryotic Genome Annotation Pipeline. Nucleic Acids Res 44:6614–6624. doi:10.1093/nar/gkw569. 27342282 PMC5001611

[10] Bortolaia V, Kaas RS, Ruppe E, Roberts MC, Schwarz S, Cattoir V, Philippon A, Allesoe RL, Rebelo AR, Florensa AF, Falgenhauer L, Chakraborty T, Neumann B, Werner G, Bender JK, Stingl K, Nguyen M, Coppens J, Xavier BB, Malhotra-Kumar S, Westh H, Pinholt M, Anjum MF, Duggett NA, Kempf I, Nykäsenoja S, Olkkola S, Wieczorek K, Amaro A, Clemente L, Mossong J, Losch S, Ragimbeau C, Lund O, Aarestrup FM. 2020. ResFinder 4.0 for predictions of phenotypes from genotypes. J Antimicrob Chemother 75:3491–3500. doi:10.1093/jac/dkaa345. 32780112 PMC7662176

[11] Jolley KA, Bray JE, Maiden MCJ. 2018. Open-access bacterial population genomics: BIGSdb software, the PubMLST.org website and their applications. Wellcome Open Res 3:124. doi:10.12688/wellcomeopenres.14826.1. 30345391 PMC6192448

[12] Wirth T, Falush D, Lan R, Colles F, Mensa P, Wieler LH, Karch H, Reeves PR, Maiden MC, Ochman H, Achtman M. 2006. Sex and virulence in *Escherichia coli*: an evolutionary perspective. Mol Microbiol 60:1136–1151. doi:10.1111/j.1365-2958.2006.05172.x. 16689791 PMC1557465

[13] Jung D, Park S, Ruffini J, Dussault F, Dufour S, Ronholm J. 2021. Comparative genomic analysis of *Escherichia coli* isolates from cases of bovine clinical mastitis identifies nine specific pathotype marker genes. Microb Genom 7:e000597. doi:10.1099/mgen.0.000597. PMC8477405 34227932

